# Wasted Holidays

**Published:** 1887-07-23

**Authors:** 


### WASTED HOLIDAYS.

The countryman needs few holidays, the townman more, the metropolitan most of all. It is impossible for a man to avoid laughing, however little of a Diogenes he may be, when he thinks, in a leisurely way, of the fuss and rattle, the irrational hurry and worry of everyday London life. What is it all about? what is it all worth? Every one of us who is now so eager to attain some end, great or small, will be for all practical purposes shut up safely in his coffin fifty years hence, to fume and fret and worry himself and his fellow-men no more. Who will care one atom then what a man thought, what he said, what he did? Whether he gained an earldom or a parish beadledom, it will be all the same.

Work is a pleasure: stern, wave-breasting, storm-defying conflict gives a keen relish to life. But over-work, worry, and mental strain are the very bane and destruction of all enduring success. It is your cool, disciplined athlete, like Grace, who scores his hundred and fifty and carries out his bat. The fussy, fuming, tearing, rushing tornado makes a great scare and sensation for a time, but seldom accomplishes anything better than the uprooting of a few venerable trees, or the blowing down of some picturesque ruin, which injured nobody and was a feature in the landscape. But whether we work wisely or foolishly, with disciplined skill or with that "raw haste" which Tennyson says is "half sister to delay," it is certain we do work, and as certain that we take it out of ourselves in a most unmistakable and often unnecessary way. Working so, we need holidays, and it is of importance to spend them in such a fashion as to get all the good they can do us, and no harm.

Some men are so undisciplined that they cannot take even their pleasures pleasantly, or enjoy leisure in a leisurely way. They are never happy unless they are on the rush, and it is difficult to imagine they are really happy even then. Such people should never take a holiday: they do not deserve one, and they certainly cannot enjoy it. The best thing they can do is to make up their minds once for all to take the bit between their teeth and tear off at full gallop, never stopping until they have galloped themselves to death. What a blessing it might be for their families if they did!

The essential characteristic of a holiday is that it be a holiday. Many people, most men perhaps, make it simply a change of business. When they are at work their business is to make money; when they take a holiday their business is to make health. They do not go holiday-making, but health-seeking. Here is a piece of advice after the manner of Poor Richard: "Take care of the holiday, and the health will take care of itself." Is the man of business so much a business man that he cannot, even for three or four weeks, throw off his business habits, and become merely a man; or better still, a good big boy? Business, whether it be commercial, legal, medical, or even clerical, is a narrow and cramping affair: a thing to be done thoroughly and well, and with zest if you like, when you are at it; but to be cast aside utterly, entirely, completely, when you take your holiday. The man should become like a boy, or a bird, or a horse in the field, or anything at all that means careless abandonment and a month of fallow.

Holidays are wasted and ruined for want of a little sense. A man says to himself, I am run down, I must go away for two or three weeks: and he goes away, perhaps to some fashionable watering-place. There he begins to take cold sea-baths in the early morning; a seven miles' walk before lunch; another six or seven miles between lunch and dinner; a kid glove and stick promenade on the pier in the evening, and then to bed, ten times more fatigued than he would have been after a hard day in his office. Next day he commences the serious business of seeing every village, every church, every old wall, every castle, ruined or otherwise, every "object of interest" or of no interest, within twenty miles of the place. He wastes his money, takes irregular meals, worries his temper, and tires himself to death by going to see what he has seen at fifty other places, and what he would not walk a single yard to see if it were within five miles of his own home. The want of imagination, of aptitude, of boyishness, of the power of easy enjoyment in the people of Great Britain is amusing and marvellous. For stolidity, stupidity, respectability, and routine, John Bull will beat the world. As it was in the beginning, is now, and ever shall be, world without end. Amen. That is his creed: and no *Athanasius contra mundum* could be more sublime in his maintenance of it, down to his very last visit to the sea-side, and his last five-guinea frock-coat for church-going wear. Most magnificent John Bull: how tremendously bovine is your attitude, and how gloriously thick your head! Does the average Briton want really to enjoy his holiday, and to come back to business like a well-trained cricketer in first-rate condition? Then let him in the first place avoid the Continent, and keep on this side the Channel. Let him go to some quiet, bracing town or village, well within reach of his means: let him resolve to be lazy, to lounge around for a few days, until he begins to feel his pulses throbbing with the stimulus of the new and purer air: let him play with his boys and girls, if he have any, or seek the woods and hills within a mile or two of his temporary home: if he can afford it, let him alternate his walks with easy drives, or an occasional excursion on the water. But why go into details? Men and women are not fools: a hint to a reasonable man is enough. The conclusion of the whole matter is this: whatever be the conventionalities, the habits, the routines which govern other people in their holidays and pleasures, as for you, when you go to the country or the sea, you will try to recall the time when you were a thoroughly careless and quite happy school-boy, and you will do your best to bring yourself and all your belongings as nearly to that condition as your family circumstances will allow. And that you may entirely succeed is the sincere wish of one who hopes he will never forget the glorious and careless days of happy boyhood. "Begone, dull care; I prithee begone from me."

